# Multi‐omic blood biomarkers and cognitive profiles discriminate causes of younger‐onset neurocognitive symptoms: a cohort study

**DOI:** 10.1002/alz.085786

**Published:** 2025-01-09

**Authors:** Oneil G Bhalala, Jessica Beamish, Patrick Summerell, Tenielle Porter, Simon M Laws, Matthew Kang, Aamira Huq, Dhamidhu Eratne, Mark Walterfang, Sarah Farrand, Andrew H Evans, Wendy Kelso, Rosie Watson, Nawaf Yassi, Dennis Velakoulis, Samantha M Loi

**Affiliations:** ^1^ Royal Melbourne Hospital, Parkville, VIC Australia; ^2^ The Walter and Eliza Hall Institute of Medical Research, Parkville, VIC Australia; ^3^ Centre for Precision Health, Edith Cowan University, Joondalup, Western Australia Australia; ^4^ Neuropsychiatry, The Royal Melbourne Hospital, Parkville, VIC Australia; ^5^ University of Melbourne, Parkville, VIC Australia; ^6^ The Royal Melbourne Hospital, Parkville, VIC Australia

## Abstract

**Background:**

Younger‐onset neurocognitive symptoms result from a heterogenous group of neurological and psychiatric disorders which present a diagnostic challenge. To identify such factors, we analysed the BeYOND (Biomarkers in Younger‐Onset Neurocognitive Disorders) cohort, a study of individuals less than 65 years old presenting with neurocognitive symptoms for a clinical diagnosis and who have undergone cognitive and biomarker analyses.

**Method:**

65 participants were recruited during their index presentation to the Royal Melbourne Hospital Neuropsychiatry Centre, a tertiary specialist service in Melbourne, Australia. Specific diagnoses were clinically determined and categorised as either early‐onset Alzheimer’s disease (AD, n=18), non‐AD neurodegeneration (nAD‐ND, n=23) or primary psychiatric disorders (PPD, n=24). Using Simoa technology, blood levels of neurofilament light chain (NfL), glial fibrillary acidic protein (GFAP), phosphorylated‐tau at threonine 181 (p‐tau181), amyloid‐β_1‐40_ (Aβ_40_), Aβ_42_ and Aβ_42/40_ ratio were measured. Apolipoprotein E (APOE) genotypes and polygenic risk scores (PRSs), based on genome‐wide association studies for late‐onset AD, were determined. Generalized linear and multinomial models, as well as information‐theoretic model selection, were used to identify discriminatory factors, adjusted for age and sex.

**Result:**

NfL, GFAP and p‐tau181 levels were up to 3‐fold higher in individuals with AD compared to nAD‐ND and PPD (Dunn‐adjusted p<0.05). Aβ‐species levels were not significantly different between the groups. While the PRS was not associated with diagnostic categories (p=0.25), a higher PRS was associated with increased NfL and GFAP levels (OR=1.08, p<0.05). Multi‐omic model selection identified various combinations of NfL and p‐tau181 levels, APOE genotype and global cognitive function as parsimonious models that discriminated between AD and PPD with AUC≥0.975 (95% CI: 0.925–1.000). A model containing only p‐tau181 significantly discriminated between AD and nAD‐ND causes (AUC=0.950, 95% CI: 0.877–1.000). Multinomial regression models identified NfL, GFAP and p‐tau181 as significant variables for discriminating between the three diagnostic categories (p<0.0005).

**Conclusion:**

Discriminating between AD, nAD‐ND and PPD causes of younger‐onset neurocognitive symptoms is possible using a combination of cognitive profiling with protein and genetic biomarkers. These results support utilising blood biomarkers for work‐up of younger‐onset neurocognitive symptoms as well as highlight the need for the development of a younger‐onset AD‐specific PRS.

